# Blood Pressure Increase and Microvascular Dysfunction Accelerate Arterial Stiffening in Children: Modulation by Physical Activity

**DOI:** 10.3389/fphys.2020.613003

**Published:** 2020-12-17

**Authors:** Giulia Lona, Christoph Hauser, Sabrina Köchli, Denis Infanger, Katharina Endes, Oliver Faude, Henner Hanssen

**Affiliations:** Department of Sport, Exercise and Health, Medical Faculty, University of Basel, Basel, Switzerland

**Keywords:** childhood health, cardiovascular risk, blood pressure, physical activity, arterial stiffness, retinal vessel diameters

## Abstract

**Background:**

Atherosclerotic remodeling starts early in life and can accelerate in the presence of cardiovascular risk (CV) factors. Regular physical activity (PA) can mitigate development of large and small artery disease during lifespan. We aimed to investigate the association of changes in body mass index (BMI), blood pressure (BP), PA behavior and retinal microvascular diameters with large artery pulse wave velocity (PWV) in prepubertal children over 4 years.

**Methods:**

The school-based prospective cohort study included 262 children initially aged 6–8 years, assessing the above CV risk factors and retinal vessels by standardized procedures at baseline (2014) and follow-up (2018). PWV was assessed by an oscillometric device at follow-up.

**Results:**

Children with increased systolic BP over 4 years showed higher PWV at follow-up (β [95% CI] 0.006 [0.002 to 0.011] mmHg per unit, *P* = 0.002). In contrast, increased vigorous PA corresponded to a lower PWV at follow-up (β [95% CI] −0.009 [−0.018 to <0−0.001] 10 min/day per unit, *P* = 0.047). Progression of retinal arteriolar narrowing and venular widening were linked to a higher PWV after 4 years (β [95% CI] −0.014 [−0.023 to −0.004] 0.01 changes per unit, *P* = 0.003).

**Conclusion:**

Increase in systolic BP and progression of microvascular dysfunction were associated with higher PWV after 4 years. Children with increasing levels of vigorous PA were found to have lower PWV at follow-up. Habitual vigorous PA has the potential to decelerate the process of early vascular aging in children and may thus help counteract CV disease development later in life.

**Clinical Trial Registration:**

ClinicalTrials.gov, Identifier: NCT03085498.

## Introduction

Regular physical activity (PA) and fitness are well known for their protective effect against cardiovascular (CV) disease development in adulthood (Lear et al., 2017). 150 min of moderate PA per week are recommended in adults (World Health Organization, 2010) and attributed to a reduced risk of CV morbidity and mortality by 22% (Lear et al., 2017). However, physical inactivity is prevalent in more than a quarter of all adults worldwide (Guthold et al., 2018) and correlates with the activity behavior in the offspring (Guthold et al., 2019). Eight of 10 children are insufficiently physically active worldwide (Guthold et al., 2019) based on the recommendation of daily 60 min moderate to vigorous PA (World Health Organization, 2010). Physical inactivity essentially contributes to the rising burden of childhood overweight and obesity (Ncd Risk Factor Collaboration, 2017) and its associated CV risk factors (Ayer et al., 2015). An increase in 1 kg/m^2^ body mass index (BMI) accounts for 1.4 mmHg higher systolic BP in prepubertal children (Falaschetti et al., 2010). Both risk factors track from childhood into adulthood (Chen and Wang, 2008; Simmonds et al., 2016) and may initiate early onset of chronic CV disease and premature CV mortality (Berenson, 2002; Franks and Bennett, 2010).

Preclinical measurements are required to determine CV health at early life exposure to initiate targeted preventive measures before clinical manifestations. Arterial stiffness is a non-invasive surrogate biomarker of the atherosclerotic process and of high clinical relevance given its predictive value of CV events and mortality in adults independent of other CV risk factors (Vlachopoulos et al., 2010). An increase in 1 m/s pulse wave velocity (PWV) is associated with a 15% higher risk for CV mortality (Vlachopoulos et al., 2010). PWV increases with age (The Reference Values for Arterial Stiffness’ Collaboration, 2010) and is related to various pathologic states, such as obesity (Liu et al., 2019), hypertension (The Reference Values for Arterial Stiffness’ Collaboration, 2010), and diabetes in adulthood (Prenner and Chirinos, 2015). A cross-talk between large and small arteries has previously been suggested, whereby CV risk factors affect both vascular beds (Nilsson et al., 2013). In children, CV risk factors have been associated with the and microvascular impairments, with a significant moderate correlation between large artery PWV and retinal microvascular diameters (Köchli et al., 2019). In adults, retinal arteriolar narrowing, venular widening and the resulting lower arteriolar to venular ratio (AVR) have been shown, for example, to predict incidence hypertension (Ikram et al., 2006b) and risk of stroke (Ikram et al., 2006a) and have been associated with cardiovascular mortality (Wang et al., 2007).

Physical activity and fitness across the life-span impede the process of arterial stiffening (Park et al., 2017), whereas most exercise intervention studies revealed an improvement in arterial stiffness accompanied by a decrease in systolic BP (Montero et al., 2014; Deiseroth et al., 2019). In childhood, higher PWV is related to increased BMI, BP, and lower cardiorespiratory fitness (CRF; Sakuragi et al., 2009; Köchli et al., 2019). Increased sedentary behavior (SB) and low levels of moderate to vigorous PA have been associated with higher arterial stiffness in childhood (Sakuragi et al., 2009; Köchli et al., 2019). The association of changes in CV risk profile, PA behavior and microvascular function with arterial stiffness in young children has not been investigated to date. We aimed to investigate the association of changes in BMI, BP, PA, and CRF as well as retinal microvascular diameters with large artery PWV as outcome after 4 years of follow-up in a prospective cohort study of prepubertal children.

## Materials and Methods

### Design and Study Population

**FIGURE 1:**
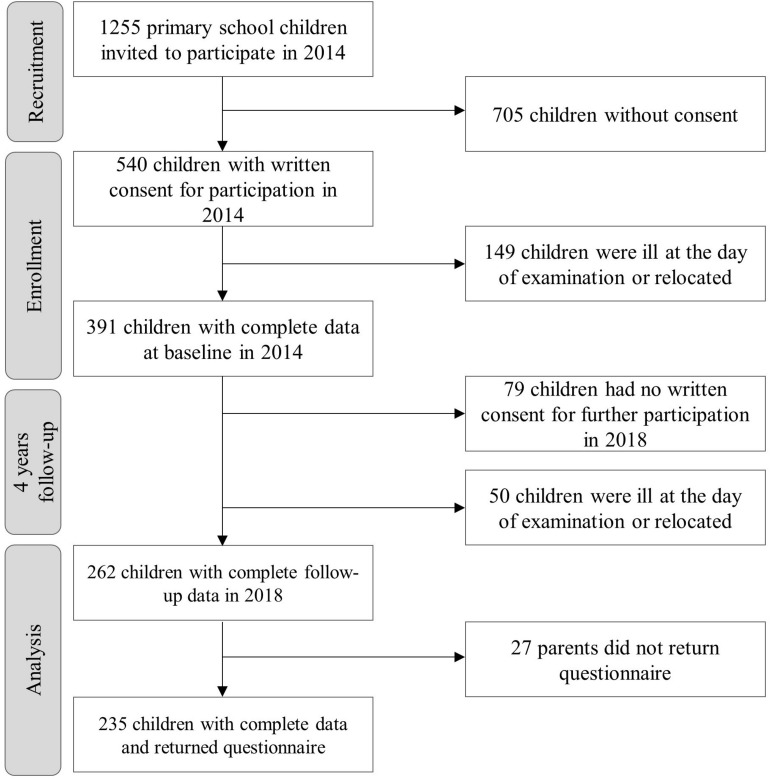
Flow chart.

The Sportcheck Follow-up study is a large cohort study in primary school children from the City of Basel, Switzerland with baseline measurements in 2014. The children were between 6–8 years old at baseline and were reexamined 4 years later in 2018. The measurements took place in school settings and were divided into medical and physical fitness assessments. To participate in the medical screening, written consent from the parents was required. The physical fitness tests were performed during regular physical education and were mandatory for all children. The recruitment and screening process of the participants is illustrated in the flow chart (Figure 1). The study was authorized by the ethics committee of North-west and central Switzerland (EKNZ, No. 258/12) and registered in a clinical trials registry (URL: http://www.clinicaltrials.gov: NCT03085498).

### Measurements

#### Pulse Wave Velocity

An oscillometric device (Mobil-O-Graph I.E.M. GmbH, Germany) was used to assess central PWV. The technique represents a simple and valid method (Franssen and Imholz, 2010; Wassertheurer et al., 2010; Weiss et al., 2012; Elmenhorst et al., 2015) with good inter- and intraobserver variability (6.3% variation; Wassertheurer et al., 2010) to monitor PWV. The appropriate cuff size was chosen based on the arm circumference and placed on the upper arm. PWV was measured in a sitting position and after a rest of 5 min to allow for central systolic BP calibration. At least two measurements were performed at 2-min intervals, which were immediately reviewed for quality and erroneous values. In case of poor data quality, further measurements were performed to be able to calculate the mean and SD of at least two valid measurements.

#### Anthropometric and Blood Pressure Measurements

Height and weight were assessed by a wall-mounted stadiometer (Seca, Basel, Switzerland) and a bioelectrical impedance analyzer (InBody 170 Biospace devise; Inbody Co, Soul, South Korea) in light clothes and without shoes. BP measurements were performed according to the guidelines of the American Academy of Pediatrics (National High Blood Pressure Education Program Working Group on High Blood Pressure in Children and Adolescents, 2004; Flynn et al., 2017). An automated oscillometric device (Oscillomate 9002, CAS Medical Systems, Branford, CT, United States) was used, previously applied in school-based intervention studies (Niederer et al., 2009; Köchli et al., 2019). Five recordings were obtained by trained personal staff and the mean of the three measurements with the smallest variation were analyzed to ensure high accuracy.

#### Cardiorespiratory Fitness and Physical Activity Behavior

A 20 m shuttle run was performed to assess CRF. It is a valid field test to estimate maximal oxygen uptake in children and adults (van Mechelen et al., 1986; Leger et al., 1988). The participants had to run back and forth between two lines 20 m apart. The running speed was paced by an audio signal and started at 8.5 km/h with an increasing speed interval of 0.5 km/h per min. The test ended when the participant was exhausted or not able to cross the line twice in a row within the given time interval. The score was assessed by the number of stages (1 stage =̂ 1 min) with a precision of 0.5 stages. PA and SB were assessed by a parental questionnaire survey. The chosen items were part of a valid PA questionnaire (Ainsworth et al., 1993; Michaud et al., 1999) and previously applied in school settings (Niederer et al., 2009; Köchli et al., 2019). Total PA (min/day) was composed of indoor and outdoor activities and sports participation (min/week) during leisure time. Vigorous PA (min/day) was defined as activity of high intensity, with the child coming out of breath or sweating. SB (min/day) included the time spent in front of a screen and comprised watching television, playing video games and using the smartphone outside of school.

#### Retinal Vessel Analysis

Static retinal vessel diameters were assessed by a fundus camera (Topcon TRC NW, Topcorn, Willich, Germany) and analyzed with the semiautomated software Visualis (Visualis 3.0, Imedos Systems UG, Jena, Germany). Two valid images from each eye were taken at an angle of 45 degrees with the optic disk at the center. Arteriolar and venular vessel diameters were identified in the area of 0.5 to 1 disk diameter from the optic disk margin. The Parr-Hubbard formula was used to average the central retinal arteriolar (CRAE) and venular (CRVE) equivalent (Hubbard et al., 1999). The AVR was quantified by the mean ratio of CRAE over CRVE.

### Statistical Analysis

To describe the population characteristic, mean and its associated confidence intervals (CI) and standard deviation was calculated for the baseline and follow-up data and compared by a simple *t*-test of dependent samples. To determine the association of BMI, BP, CRF, PA, and retinal vessel diameters with PWV, a mixed linear regression model was performed considering schools as random effects (Twisk, 2006; West et al., 2014). The assumptions of normal distribution and heteroscedasticity of the residuals were checked graphically (Brown and Prescott, 2015). The first model was adjusted for the covariates of age, sex and height at baseline. The second model was extended by the covariates of weight and BP at baseline. The above-mentioned statistical procedure was also used to investigate the association of changes in BMI, BP, physical fitness, activity behavior and retinal vessel diameters with PWV. The change of the dependent variable over 4 years was calculated as the difference from baseline to follow-up. The regression models were presented in β coefficients and its associated 95% CIs. A sample size calculation was conducted *a priori* based on the previously reported association of BP and retinal vessel diameters (Lona et al., 2020). A power of about 95% was estimated with a sample of 250 children (Lona et al., 2020). A significance level of 0.05 was used and all calculations were performed with Stata 15 (StataCorp, College Station, TX, United States).

## Results

### Population Characteristics

In total, 391 children were included at baseline aged 6–8 years and, 4 years later, 262 children were reexamined. 50 children were ill or relocated at the day of examination. The lost to follow-up rate accounts for 33%. Participants demographics at baseline and follow-up are presented in Table 1. The children at 4 years follow-up evolved significantly higher BMI (Δ 2.0 ± 1.7 kg/m^2^), BP (Δ systolic BP 4.0 ± 8.3 mmHg; Δ diastolic BP 1.7 ± 7.6 mmHg), and CRF (Δ 1.8 ± 1.7 stages) within childhood development. Regarding daily PA, the participants were less engaged in total PA (Δ 45.1 ± 83.3 min/day) and spent more time sedentary (Δ 32.7 ± 50.3 stages) at follow-up. However, the time spent in vigorous PA remained stable from baseline to follow-up with approximately 55 min per day. In comparison to baseline, narrower CRAE (Δ−6.3 ± 8.6 μm) and no significant changes in CRVE (Δ−0.2 ± 7.9 μm) were detected at follow-up. A mean PWV of 4.7 ± 0.3 m/s was observed at the age of 10–12 years.

**TABLE 1:** Population characteristics.

	**Baseline**			**4 years follow-up**		**Difference**		
	***N***	**Mean**	**SD**	**Mean**	**SD**	**Mean**	**SD**	***P*-value**
Sex (male,%)	262	45.8						
Age (y)	262	7.4	0.3	11.4	0.3	4.0		
Height (m)	248	1.3	0.1	1.5	0.1	0.2	0.3	<0.001
Weight (kg)	248	25.5	4.0	40.3	8.4	14.8	5.3	<0.001
BMI (kg/m^2^)	248	16.0	1.9	18.1	3.0	2.0	1.7	<0.001
Z-BMI*	260	−0.1	0.9	−0.3	1.0	0.2	0.6	<0.001
Systolic BP (mmHg)	260	104.4	7.4	108.3	7.8	4.0	8.3	<0.001
Z-systolic BP*	260	0.6	0.9	1.6	0.9	1.0	1.0	<0.001
Diastolic BP (mmHg)	260	65.3	7.0	67.0	6.9	1.7	7.6	<0.001
Z-diastolic BP	258	0.6	1.1	1.3	1.0	0.7	1.1	<0.001
Central systolic BP (mmHg)	262			99.9	8.0			
Central diastolic BP (mmHg)	262			73.9	6.9			
Central pulse pressure	262			26.4	5.0			
CRAE (μm)	262	206.4	14.0	200.1	13.1	−6.3	8.6	<0.001
CRVE (μm)	262	231.8	12.8	231.6	13.3	−0.2	7.9	0.740
AVR	262	0.89	0.5	0.87	0.6	−0.03	0.4	<0.001
PWV (m/s)				4.7	0.3			
CRF (stages)	235	4.6	1.7	6.4	2.0	1.8	1.7	<0.001
MVPA (min/day)	192	149	64	104	66	1.8	44.6	0.610
Total PA (min/day)	196	44	44	76.7	62	45.1	83.3	<0.001
SB (min/day)	235	4.6	1.7	6.4	2.0	32.7	50.3	<0.001

* Z-BMI, Z-systolic BP, and Z-diastolic BP are based on the KiGGS (German Health Interview and Examination Survey for Children and Adolescents) reference values. AVR indicates arteriolar-to-venular diameter ratio; BMI, body mass index; BP, blood pressure; CRAE, central arteriolar equivalent; CRVE, central venular equivalent; CRF, cardiorespiratory fitness (1 stage =̂ 1 min); MVPA, moderate to vigorous physical activity; PA, physical activity; PWV, pulse wave velocity; SB, sedentary behavior; and SD, standard deviation.

### Association of Changes in CV Risk Profile With Arterial Stiffness at Follow-Up

Table 2 presents the association of changes in BMI, BP, and retinal vessel diameters over 4 years with arterial stiffness at follow-up. Increases in BMI over 4 years were not significantly related to a higher PWV at follow-up (β [95% CI] 0.002 [−0.019 to 0.023] m/s per 1 kg/m^2^ increase, *P* = 0.860). The comparison of weight categories also revealed little evidence (*p*-value of an independent *t*-test: 0.29) that children with overweight or obesity (mean [95% CI] 4.72, [4.6–4.8] m/s) at baseline had a higher PWV at follow-up compared to their lean peers (mean [95% CI] 4.7 [4.6–4.7] m/s; Supplementary Figure 1). Increase in systolic BP was significantly related to a higher PWV (β [95% CI] 0.006 [0.002 to 0.011] m/s per 1 mmHg increase, *P* = 0.002) at follow-up. A 0.01 unit decrease of retinal AVR over 4 years was associated with a higher PWV by 0.014 m/s (β [95% CI] 0.014 [−0.023 to −0.004] per 0.01 unit decrease in AVR, *P* = 0.003), reflected by microvascular narrowing of CRAE (β [95% CI] −0.055 [−0.094 to −0.015] m/s per 10 μm decrease, *P* = 0.007).

**TABLE 2:** Association of changes in body mass index, blood pressure, and retinal vessel diameters with pulse wave velocity at follow-up.

		**Pulse wave velocity at follow-up (Increase per 1 m/s)**	
**Parameter**	**Model**	**B (95% CI)**	***P*-value**
Δ BMI (kg/m^2^ increase per unit)	1	0.002 (−0.019 to 0.023)	0.860
	2	−0.005 (−0.027 to 0.017)	0.651
Δ Systolic BP (mmHg increase per unit)	1	0.006 (0.002 to 0.011)	**0.002**
	3	0.006 (0.002 to 0.011)	**0.002**
Δ Diastolic BP (mmHg increase per unit)	1	0.004 (−0 to 0.009)	0.067
	3	0.004 (−0 to 0.009)	0.056
Δ CRAE (10 μm increase per unit)	1	−0.055 (−0.094 to −0.015)	**0.007**
	2	−0.014 (−0.04 to 0.012)	0.292
Δ CRVE (10 μm increase per unit)	1	0.004 (−0.04 to 0.048)	0.844
	2	<−0.001 (−0.043 to 0.041)	0.957
Δ AVR (0.01 increase per unit)	1	−0.014 (−0.023 to −0.005)	**0.003**
	2	−0.013 (−0.022 to −0.004)	**0.004**

*Model 1: adjusted for age, sex, and height. Model 2: adjusted for model 1 plus weight, systolic BP and diastolic BP. Model 3: adjusted for model 1 plus weight. AVR indicates arterio-to venular ratio; BP, blood pressure; Confidence Interval, CI; CRAE, central arteriolar equivalent; and CRVE, central venular equivalent. Bold numbers indicate significant p-values.*

### Association of Changes in Cardiorespiratory Fitness and Physical Activity Behavior With Arterial Stiffness at Follow-Up

The association of CRF, PA behavior with PWV at follow-up are demonstrated in Table 3. In contrast, increase of time spent with vigorous PA was associated with decreased PWV (β [95% CI] –0.009 [−0.018 to <−0.001] m/s per 10 min increase, *P* = 0.047), independent of BP and weight (β [95% CI] −0.021[−0.021 to −0.003] m/s per 10 min increase, *P* = 0.010). However, increase in CRF levels over 4 years did not correlate with lower PWV (β [95% CI] 0.005 [−0.017 to 0.027] m/s per 1 stage increase, *P* = 0.665) at follow-up. Interestingly, the subgroup analysis of children with elevated or high systolic BP at baseline (*n* = 62) revealed a significant lower PWV at follow-up in those children who increased their total PA (β [95% CI] −0.010 [−0.021 to −<0.001] m/s per 10 min increase, *P* = 0.048) and not vigorous PA. No association between increases in total PA (β [95% CI] <−0.001 [−0.001 to 0.001] m/s per 10 min increase, *P* = 0.910) or vigorous PA (β [95% CI] −0.002 [−0.002 to 0.001] m/s per 10 min increase, *P* = 0.843) and PWV at follow-up was found in children with overweight or obesity.

**TABLE 3:** Association of changes in cardiorespiratory fitness and physical activity behavior with pulse wave velocity at follow-up.

		**Pulse wave velocity at follow-up (Increase per 1 m/s)**	
**Parameter**	**Model**	**B (95% CI)**	***P*-value**
Δ Cardiorespiratory fitness (stages increase per unit)	1	0.005 (−0.017 to 0.027)	0.665
	2	0.008 (−0.013 to 0.029)	0.477
Δ Vigorous physical activity (10 min/day increase per unit)	1	−0.009 (−0.018 to <−0.001)	**0.047**
	2	−0.012 (−0.021 to −0.003)	**0.010**
Δ Total physical activity (10 min/day increase per unit)	1	0.002 (−0.003 to 0.007)	0.378
	2	0.001 (−0.003 to 0.006)	0.539
Δ Sedentary Behavior (10 min/day increase per unit)	1	<0.001 (−0.008 to 0.008)	0.995
	2	−0.001 (−0.009 to 0.006)	0.766

*Model 1: adjusted for age, sex, and height. Model 2: adjusted for model 1 plus weight, systolic and diastolic blood pressure. Cardiorespiratory fitness (1 stage =̂ 1 min). Bold numbers indicate significant p-values.*

## Discussion

We investigated the association of 4 years changes in BMI, BP, PA behavior and CRF as well as retinal microvascular diameters with large artery PWV at follow-up in 262 prepubertal children. Increase in systolic BP during the observation period was associated with higher PWV at follow-up. A lower AVR over 4 years, reflected by retinal arteriolar narrowing was related to a higher PWV. In contrast, an increase in vigorous PA over 4 years contributed to a favorably lower PWV at follow-up.

The results of our study indicate an positive association between prospective increase in systolic BP and PWV at follow-up. These results are in line but go beyond our previous report of an inverse correlation between BP and PWV at the cross-sectional level (Köchli et al., 2019). In childhood, increase in BP seems to be driven by higher sympathetic nervous system activity and peripheral resistance resulting in higher arterial stiffness (Thorp and Schlaich, 2015; Aroor et al., 2018). At a later stage, manifestation of high BP might be governed by arterial stiffness rather than the sympathetic nervous system (Thorp and Schlaich, 2015; Aroor et al., 2018). However, BP and PWV are tightly linked to each other and in adults, it is an ongoing debate whether arterial stiffness is a cause or a consequence of hypertension, which may evolve in a vicious cycle (Mitchell, 2014; Safar et al., 2018). Nevertheless, PWV has shown to be of added value in the risk prediction of CVD development and mortality independent of blood pressure (Vlachopoulos et al., 2010). The Bogalusa heart study demonstrated that systolic BP in childhood is predictive for increased arterial stiffness in early adulthood (Li et al., 2004). The study of Young Finns revealed that a favorable change from elevated BP in childhood to normal BP in adulthood was related to a lower PWV in young adults, indicating modification of atherosclerotic risk by BP decreases in early adulthood (Aatola et al., 2017).

Our findings demonstrate no significant association between BMI at baseline and PWV at follow-up. In 9 years old children, body fat but not BMI was related to increased arterial stiffness in mid-adolescence (Dangardt et al., 2019). An adverse association between BMI and PWV in adulthood has been shown to appear in late adolescence (Ferreira et al., 2012). The temporal relationship of BMI and arterial stiffness in childhood might underly the complexity of accelerated growth and early onset of pubertal maturation in children with overweight and obesity (Tryggestad et al., 2012). Short-term adaptations of vascular dilatation and expedited vascular network growth are assumed to protect the vasculature against increased mechanical stress (Tryggestad et al., 2012) and might partly explain the lack of a longitudinal association between BMI and arterial stiffness in prepubertal children.

With respect to the cross-talk between the macro- and microvascular beds, narrowing of CRAE and widening of CRVE over 4 years, resulting in an unfavorable lower AVR, were related to a higher PWV at follow-up. However, the association of CRAE with PWV was not independent of BP and no direct association between CRVE and PWV was found. It was, therefore, the low retinal AVR which was inversely and independently associated with higher PWV at follow-up. From a physiological perspective, the BP-dependent modulation of the cross-talk between microvascular CRAE and large artery PWV seems comprehensible. In a simple model, blood flow, determined by pressure gradient and vascular resistance, is the decisive link between the micro- and macrovascular bed (Safar and Struijker-Boudier, 2010). The macrocirculation generates pulsatile pressure and flow, whereas the microcirculation requires constant pressure and continuous flow. The pulse wave is reflected in the microcirculation and thus augments the pressure wave and affects the pulse pressure of large arteries. In turn, an increased pulse pressure as a consequence of increased arterial stiffness impairs the microvascular system, with further increase in peripheral resistance, with the vicious cycle potentially causing long-term target-organ damage (Safar and Struijker-Boudier, 2010). Our results suggest, that this unfavorable cycle may develop as early as childhood.

On the other hand, increase in vigorous PA over 4 years was related to lower arterial stiffness at follow-up independent of weight and blood pressure. The amount spent in total PA was not associated with improved arterial stiffness except for the subgroup of children with either elevated or high BP. However, this finding has to be verified in a larger cohort of children with increased risk of high blood pressure. Vigorous PA increases blood flow and laminar shear stress and leads to an increased nitric oxide production and pronounced vasodilation. An increased stimulus of shear stress and nitric oxide bioavailability over time, induced by vigorous PA, may lead to a favorable vascular wall integrity and may explain why increases in total PA were not sufficient to improve PWV at follow-up. A detailed investigation of the underlying mechanisms was beyond the scope of this study. Similar results were found in preschool children, indicating that higher levels of moderate to vigorous PA can contribute to a slower age-related progression of PWV (Proudfoot et al., 2019). The retrospective study by van de Laar et al. (2010) demonstrated that increased vigorous PA from puberty to adulthood had a favorable impact on arterial stiffness in young adults. However, the association was not independent of CV risk factors and the effect of arterial stiffness on changes in PA behavior during lifespan was not investigated (van de Laar et al., 2010).

We found little evidence for an association between changes in CRF at baseline and arterial stiffness at follow-up. In childhood, more than half of the individual differences in maximal oxygen uptake are determined by genetic factors (Schutte et al., 2016). Heritability might explain the lack of association between CRF and arterial stiffness over 4 years and is supported by the findings of the large Amsterdam Growth and Health Longitudinal Study. The study investigated whether CRF from adolescence to young adulthood (13–36 years) determined arterial stiffness in 36 years old individuals (Ferreira et al., 2012). Significant differences between groups of low and high arterial stiffness were detected by the level of CRF performance from the age of 27 and not earlier (Ferreira et al., 2012).

Some limitations need to be addressed. 33% of the included participants did not participate in the follow-up measurements due to illness at the day of examination, relocation or personal reasons. However, the population characteristics of the loss to follow-up group did not differ notably form the follow-up group (Supplementary Table 1) and, thus, the risk of a selection bias was considered to be small. PWV was only assessed at follow-up and in children age 10–12 years and, therefore, adverse causality cannot be ruled out. Furthermore, we were not able to analyze PWV at the same pressure level to identify intrinsic vascular alterations. In our analyses, we did, however, adjust for BP and the presented findings were independent of BP. Our subgroup analysis in children with elevated or high systolic BP and the impact of changes in total PA on PWV includes only a relatively small number of participants. Future research in a larger cohort is required to verify our findings.

The clinical relevance of quantifying arterial stiffness in childhood is unclear and requires future research to establish evidence-based recommendations for the assessment of arterial stiffness in children. Furthermore, we carefully assessed BP status with five separate measurements for each participant. However, the measurements were taken on a single occasion and did not allow to identify children with hypertension or white coat hypertension. PA behavior was assessed by a parental questionnaire and entails the risk of response and recall bias. Nevertheless, the chosen method allowed to measure a large sample size and we achieved a relatively high response rate of 80% (*n* = 235). Two retinal vessel images of each eye were semi-automatically analyzed to guarantee high accuracy. Reference values for the retinal vessel diameters in children and adults are not available yet.

## Perspectives

Increase in systolic BP over 4 years was associated with higher arterial stiffness at follow-up. Progression of microvascular alterations from baseline to follow-up was related to higher arterial stiffness. However, our results demonstrate that increases in vigorous PA may counteract development of large artery stiffness in prepubertal children. Vigorous habitual PA in childhood has the potential to decelerate the process of vascular aging and to reduce the burden of CV disease development later in life.

## Data Availability Statement

The original contributions presented in the study are included in the article/Supplementary Materials, further inquiries can be directed to the corresponding author/s.

## Ethics Statement

The studies involving human participants were reviewed and approved by Ethics committee of North-west and central Switzerland (EKNZ, No. 258/12). Written informed consent to participate in this study was provided by the participants’ legal guardian/next of kin.

## Author Contributions

GL conceptualized and designed the study, collected data, performed the statistical analysis, prepared, and revised the manuscript. CH and SK revised the manuscript. DI performed the statistical analysis, reviewed, and revised the manuscript. KE conceptualized and designed the study and revised the manuscript. OF critically reviewed the manuscript. HH conceptualized and designed the study, helped perform the statistical analysis, prepared, and critically reviewed the manuscript. All authors contributed to the article and approved the submitted version.

## Conflict of Interest

The authors declare that the research was conducted in the absence of any commercial or financial relationships that could be construed as a potential conflict of interest.
