# Supplementary material for: Blood Pressure Increase and Microvascular Dysfunction Accelerate Arterial Stiffening in Children: Modulation by Physical Activity
Source: Front Physiol. 2020 Dec 17;11:613003. doi: 10.3389/fphys.2020.613003 (PMC7773656; doi:10.3389/fphys.2020.613003)
Supplement: Supplementary file 1 [file Data_Sheet_1.DOCX]

Supplementary Material

Table S1: Baseline population characteristics in children with and without follow-up

AVR indicates arteriolar-to-venular diameter ratio; BMI, body mass index; BP, blood pressure; CI, Confidence Interval; CRAE, central arteriolar equivalent; CRVE, central venular equivalent; CRF, cardiorespiratory fitness; PA, physical activity; SB, sedentary behavior; SD, standard deviation

| Parameter | | With Follow-up | | |  | Without Follow-up | | |  | Differences | |
| --- | --- | --- | --- | --- | --- | --- | --- | --- | --- | --- | --- |
|  |  | N | Mean | SD |  | N | Mean | SD |  | Mean  (95% CI) | P-Value |
| Sex (male, %) |  | 120 | 46.1 |  |  | 85 | 49.4 |  |  |  |  |
| Age (y) |  | 260 | 7.4 | 0.3 |  | 172 | 7.4 | 0.3 |  |  |  |
| Height (cm) |  | 259 | 126.0 | 5.0 |  | 171 | 126.0 | 5.9 |  | 0.03  (-1.02 to 1.07) | 0.962 |
| Weight (kg) |  | 259 | 25.7 | 4.1 |  | 171 | 26.4 | 5.2 |  | 0.7  (-0.16 to 1.59) | 0.121 |
| BMI (kg/m^2^) |  | 259 | 16.1 | 1.9 |  | 171 | 16.5 | 2.3 |  | 0.39  (-0.01 to 0.79) | 0.057 |
| Systolic BP (mmHg) |  | 258 | 104.4 | 7.5 |  | 168 | 104.8 | 8.6 |  | 0.44  (-1.11 to 1.99) | 0.578 |
| Diastolic BP (mmHg) |  | 258 | 65.3 | 7.0 |  | 168 | 65.9 | 6.7 |  | 0.56  (-0.78 to 1.90) | 0.410 |
| CRAE (µm) |  | 260 | 206.4 | 14.1 |  | 172 | 204.7 | 14.0 |  | -1.69  (-4.41 to 1.02) | 0.221 |
| CRVE (µm) |  | 260 | 231.9 | 12.8 |  | 172 | 231.4 | 14.4 |  | -0.43  (-3.03 to 2.17) | 0.745 |
| AVR |  | 260 | 0.89 | 0.05 |  | 172 | 0.89 | 0.05 |  | -0.005  (-0.01 to <0.01) | 0.275 |
| CRF (stages) |  | 250 | 4.5 | 1.7 |  | 168 | 4.2 | 1.6 |  | -0.38  (-0.71 to -0.06) | **0.019** |
| PA (min/week) |  | 230 | 217.6 | 123.0 |  | 137 | 207.6 | 115.2 |  | -9.96  (-35.50 to 15.50) | 0.443 |
| SB (min/week) |  | 234 | 46.5 | 44.4 |  | 144 | 53.5 | 51.0 |  | 6.99  (-2.81to 16.78) | 0.162 |

**Figure S1:**


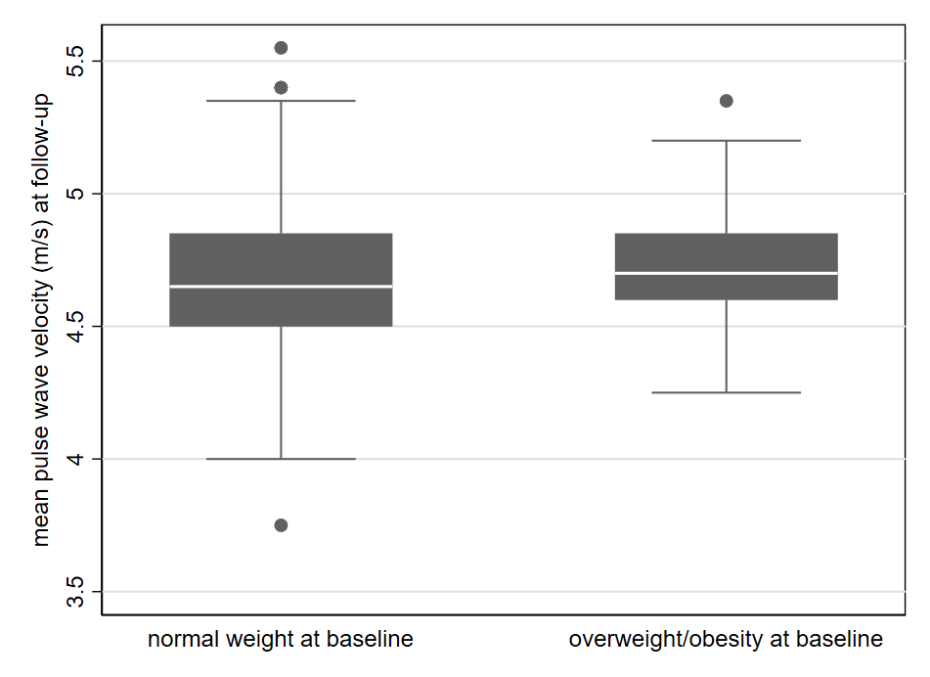


Pulse wave velocity at follow-up in children with normal weight and overweight or obesity at baseline. Values are

expressed as median and quartile. P-value of an independent t-test between groups: 0.2898.
